# Benzodiazepine Delorazepam Induces Locomotory Hyperactivity and Alterations in Pedal Mucus Texture in the Freshwater Gastropod *Planorbarius corneus*

**DOI:** 10.3390/ijms242317070

**Published:** 2023-12-02

**Authors:** Chiara Fogliano, Rosa Carotenuto, Claudio Agnisola, Palma Simoniello, Myriam Karam, Claudia Manfredonia, Bice Avallone, Chiara Maria Motta

**Affiliations:** 1 Department of Biology, University of Naples Federico II, 80126 Naples, Italy; chiara.fogliano@unina.it (C.F.); rosa.carotenuto@unina.it (R.C.); agnisola@unina.it (C.A.); m.karam@studenti.unina.it (M.K.); cla.manfredonia@studenti.unina.it (C.M.); mottacm@unina.it (C.M.M.); 2 Department of Science and Technology, University of Naples Parthenope, 80143 Naples, Italy; palma.simoniello@uniparthenope.it

**Keywords:** locomotory test, feeding behavior, pedal glands, mucus texture, foot protein pattern, lectin staining

## Abstract

Benzodiazepines, psychotropic drugs, are ubiquitous in the aquatic environment due to over-consumption and inefficient removal by sewage treatment plants. Bioaccumulation with consequent behavioral and physiological effects has been reported in many aquatic species. However, the responses are species-specific and still poorly understood. To improve the knowledge, we exposed the freshwater snail *Planorbarius corneus* to 1, 5, or 10 µg/L of delorazepam, the most widely consumed benzodiazepine in Italy. Conventional behavioral tests were used to assess the effects on locomotor and feeding behavior. Histological and biochemical analyses were also performed to detect possible changes in the structure and composition of the foot mucus and glands. The results show a paradoxical response with reduced feeding activity and locomotor hyperactivity. Pedal mucus was altered in texture but not in composition, becoming particularly rich in fibrous collagen-like material, and a significant change in the protein composition was highlighted in the foot. In conclusion, exposure to delorazepam induces disinhibited behavior in *Planorbarius corneus*, potentially increasing the risk of predation, and an increase in mucus protein production, which, together with reduced feeding activity, would severely compromise energy resources.

## 1. Introduction

Since the 1960s, benzodiazepines (BZDs) have been rapidly commercialized worldwide since replacing older sedatives and hypnotics and were initially welcomed with enthusiasm [1]. However, concerns rapidly arose about the risk of dependence. Nowadays, BZDs are among the primary substances of abuse [2] and among the most frequently found pharmaceutical residues (active principle and metabolites) in aquatic environments [3,4,5,6,7,8]. This happens because wastewater treatment plants (WWTPs) are generally designed to handle easily degradable organics and therefore not conceived to treat pharmaceuticals, which is why they are not efficient in their removal. Remediation efficiencies can be less than 10% for some pharmaceutical classes, BZDs among these [9,10]. As an inevitable consequence, BZD residues reach the aquatic environment and, being resistant to photodegradation [11], persist for decades, accumulating in the food chain [12,13]. Their stability in the environment has been demonstrated, with persistence at unchanged concentrations in lake sediments for several decades [14]. The activity of these drugs is of considerable concern, as they are specifically synthesized to elicit responses in humans and animals even at low concentrations [15], representing, therefore, a hazard, especially for aquatic species with which they inevitably come into contact. Concentrations from nanograms to several micrograms have been reported in surface waters [10,11], a range in which measurable effects on target organisms can actually be exerted: GABA-A receptors are evolutionarily conserved across the animal kingdom [16], and their improper activation induces relevant behavioral alterations. Marked depression of motor activity is registered, for example, in *Hydra*, *Daphnia*, and *Dugesia* [17] or *Radix balthica* [18]. However, exposure is also reported to stimulate locomotor performance, a paradoxical effect reported, for example, in *Danio rerio* [19]. BZD effects, therefore, may vary in a species-specific manner, but evidence also indicates differences in response according to the dose (*Xenopus laevis* embryos; [20]) or type of treatment, acute or chronic (*Hediste diversicolor*, [21]). In addition, it has been demonstrated that BZDs may exert dissimilar effects on different forms of behaviors, for example, on feeding and swimming [20,22,23]. Moreover, a possible direct effect on tissues should be considered: BZDs in fact can also exert their action via the peripheral benzodiazepine receptors (TSPO) located on mitochondrial membranes [24], which regulate cell energy metabolism, transmembrane potential, and sensitivity to reactive oxygen species [25]. TSPOs are ubiquitously expressed in all tissues and are known to take part in CNS pathological disorders but also to play a key role in neuronal apoptosis, glial cell degeneration, and regeneration [26]. 

Based on these considerations, in the present study, we analyzed the effects of the benzodiazepine delorazepam (DLZ) on locomotion in the freshwater gastropod *Planorbarius corneus*. This is a common model in ecotoxicological studies [27,28], as living in freshwater exposes it to many water pollutants. Three increasing concentrations were tested, considering the increase in BZD concentration in water in the last decade [29,30] and that the COVID-19 pandemic further contributed to reaching a peak [31,32]. Locomotory behavior was first assayed by determining the interference of thigmotactic and food search response activity [33,34], common endpoints in ecotoxicological studies, routinely employed in different animal species [35,36,37,38]. In gastropods, the test has been used in Cerithiids, to study aggregation models [39] and in *Littorina* to decipher unknown aspects of behavioral ecology [40]. Gastropods move by gliding on the substrate, an activity dependent on the combination of mucus release (forming a 10–20 µm thick layer; [41]), pedal muscle activity, and the metachronal beating of the sole’s epithelial cells cilia. Velocity is controlled by the viscoelastic properties of the mucus that tends to expand in water, forming a gel [42,43], also granting adhesion to the substrate [41]. Considering that DLZ may alter locomotion, we also investigated possible changes in mucus texture and composition. Content in glycoconjugates was determined by PAS and Alcian blue, while a panel of three lectins (WGA, UEA-I, DBA) highlighted sugar residue presence and abundance [44]. Cytological investigations were extended to pedal glands responsible for mucus release. The presence of collagen [45] was detected by staining with Picrosirius red [46], while the effects on foot protein patterns were investigated by SDS-PAGE. 

## 2. Results

### 2.1. Effects of Delorazepam on Locomotor Activity

**Figure 1:**
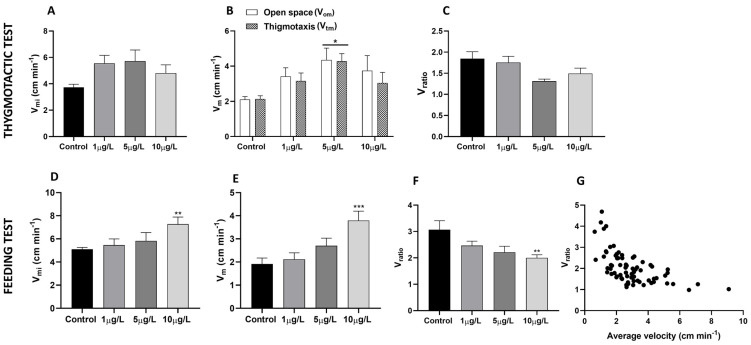
Response to thigmotactic and feeding test in Planorbarius corneus exposed to DLZ 1, 5, or 10 μg/L. (**A**) In the thigmotactic test, a slight increase was detected in mean instantaneous velocity (V_mi_). (**B**) Significant increase in mean velocity in 5 μg/L treated animals both in open space and during thigmotaxis (two-way ANOVA followed by the Sidak test; * *p* < 0.05). (**C**) Moderate but not significant decrease in Vratio (=V_mi_/V_m_). (**D**,**E**) In the feeding test, dose-dependent increase in mean instantaneous velocity (V_mi_) and mean velocity (Vm) become significant in 10 μg/L treated (one-way ANOVA, followed by the Tukey test, ** *p* < 0.01, *** *p* < 0.001). (**F**) Dose-dependent decrease in Vratio, with significance in 10 μg/L treated (one-way ANOVA, followed by Tukey test, ** *p* < 0.01). (**G**) Correlation between Vratio and Vm, obtained by pooling data from both behavioral tests. Vratio tends to be 1 when the speed exceeds 5 cm/min. The correlation test is highly significant (r^2^ = 0.3676, *p* < 0.0001, N = 80).

*P. corneus* locomotor activity is stimulated by exposure to DLZ. In both the thigmotactic (Figure 1A–C) and feeding tests (Figure 1D–F), the mean instantaneous velocity V_mi_ (Figure 1A,D) moderately increases. More relevant is the effect observed on the average speed V_m,_ which increases very significantly in animals treated with DLZ 5 µg/L (thigmotactic test) and 10 µg/L (feeding test). In the thigmotactic test, the increase is observed both in open space (V_om_) and along the wall of the arena (V_tm_). In contrast, the ratio between the mean instantaneous velocity and mean velocity (V_ratio_, Figure 1C,F) tends to decrease, and the most significant data are registered in the feeding test at 10 μg/L. The decreasing trend of the V_ratio_ indicates the tendency of the animals to follow a less tortuous path while they move faster. As shown in Figure 1G, this ratio tends to be 1 when the speed exceeds 5 cm/min.

### 2.2. Effects of Delorazepam on Feeding Behavior Response

**Figure 2:**
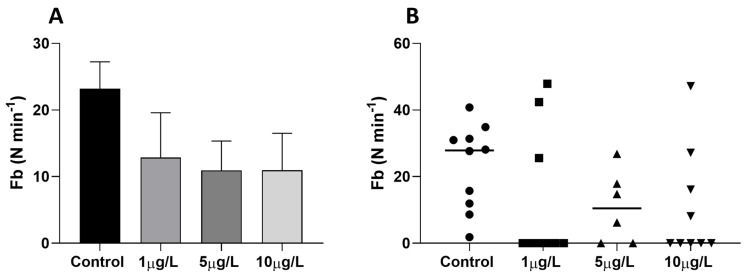
Feeding behavior in Planorbarius corneus exposed to DLZ 1, 5, or 10 μg/L. (**A**) Feeding activity, expressed as the number of mouth openings (Fb) per minute, was determined as the animal explored the chamber. Mean values ± SD. (**B**) Distribution of individual values around the median; in the treated groups, differently from the control, a significant number of individuals did not display buccal activity during the trial (binomial test, *p* < 0.0001).

In the food search test, all control animals showed a continuous and intense exploratory activity of the mouth apparatus that opens an average of 24 times per minute (Figure 2A). In DLZ-treated animals, at each dose tested, the average number of openings is reduced since a significant number of animals (Figure 2B) do not open their mouths at all. In addition, despite reaching the food, these individuals avoid it rather than starting to eat. A correlation analysis carried out between the frequency of opening of the buccal apparatus and the average speed showed that there is no correlation in the controls (r^2^ = 0.155, *p* = 0.260, N = 10) and the group treated with 5 µg/L (r^2^ = 0.137, *p* = 0.470, N = 6). In contrast, in groups treated with 1 and 10 µg/L, the buccal activity is inversely related to speed (1 µg/L: r^2^ = 0.469, *p* = 0.042, N = 9; 10 µg/: r^2^ = 0.769, *p* = 0.042, N = 9).

### 2.3. Effects of Delorazepam on Mucus Texture

**Figure 3:**
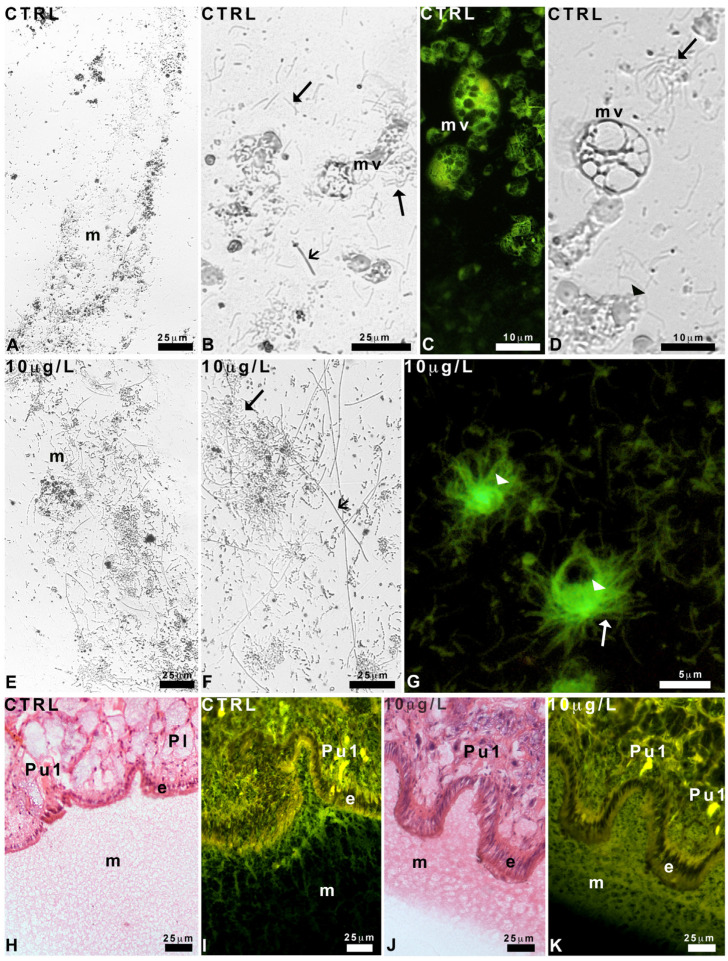
Texture of the mucus released by Planorbarius corneus, control or exposed to delorazepam 10 µg/L. (**A**–**G**) Trails. (**H**–**K**) Histological sections. (**A**,**B**) Overview of mucus (m). Note the presence of a multi-vesiculated body (mv), thick filaments (small arrow), and thin filaments (arrow). (**C**) Detail of multi-vesiculated bodies (mv). (**D**) Detail of a multi-vesiculated body (mv), a tangle of thin filaments (arrow), and round bodies (arrowhead). (**E**,**F**) Overview of mucus (m), thin (arrow), and thick filaments (small arrow). (**G**) Eosin-stained round bodies (arrowhead) and the thin filaments extruding from it (arrow). (**H**,**I**) Homogeneous mucus (m) with a finely reticular texture. Epithelium (e), pedal glands (Pl and Pu1). (**J**,**K**) Compact, bi-layered mucus (m). Epithelium (e), pedal glands (Pl and Pu1). Phase contrast (**A**,**B**,**D**–**F**). Hemalum-eosin staining: bright field (**H**,**J**), UV light (**C**,**G**,**I**,**K**).

Control animals leave behind a trail containing epithelial residues, filaments, and vesicles (Figure 3A–D). Filaments are either thick and quite short or thin and abundant, with a marked tendency to form bunches (Figure 3B). Vesicles may be large and “multi-vesiculated” (Figure 3C,D), containing dense material and intensely fluorescent under UV light (Figure 3C). Poorly eosinophilic round bodies are also identifiable (Figure 3D).

After exposure to DLZ 10 µg/L, the trails appear denser (Figure 3E,F); the thick filaments are numerous, straight, extremely long (up to 750 µm), and intermingled with less numerous thin filaments (Figure 3G). Multi-vesiculated bodies are rare; in contrast, vesicles containing thin, eosin-positive filaments are very numerous (Figure 3G). 

In histological sections, the mucus stains intensely with eosin, confirming the presence of a conspicuous protein component. In control animals (Figure 3H), it is homogeneous, with a finely reticular texture. Observation under UV light, which detects the autofluorescence of the eosin stain, confirms the loose reticular texture (Figure 3I). 

No significant differences with respect to the control are observed in the feet of snails exposed to 1 µg/L or 5 µg/L of DLZ. In contrast, in animals exposed to DLZ 10 µg/L, the mucus layer appears thinner and more compact, with a clear-cut external contour (Figure 3J). UV light confirms the change in texture and suggests the existence of a bi-layered organization (Figure 3K). 

### 2.4. Pedal Glands and Role in Mucus Release

**Figure 4:**
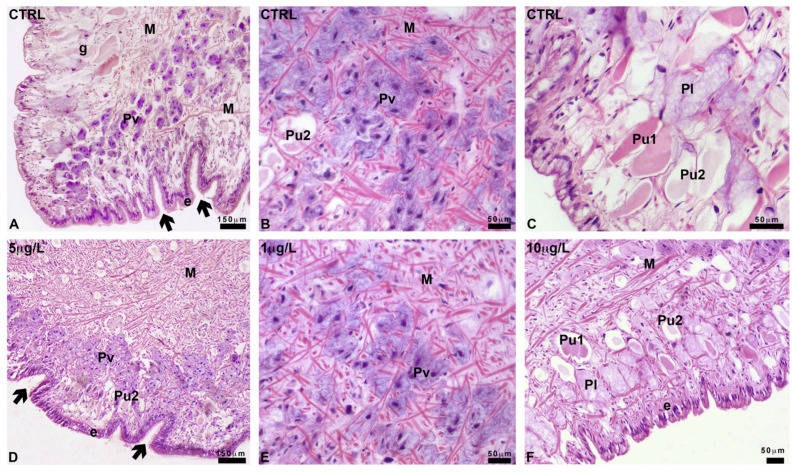
Foot gland anatomy in Planorbarius corneus, control or exposed to delorazepam. (**A**) Sole infoldings (thick arrows) lined with columnar epithelium (e). Ventral gland cells (Pv) and basolateral area with glands (g). Muscle fiber (M). (**B**) Ventral gland cells (Pv) stained by hemalum; ubiquitary Pu2 gland and muscle fibers (M). (**C**) Basolateral region; closely packed lateral (Pl) and ubiquitary (Pu1 and Pu2) glands. (**D**) Foot sole with infoldings (thick arrow), epithelium (e), ventral (Pv), and ubiquitary Pu2 glands. Muscle fibers (M). (**E**) Ventral gland cells (Pv) stained by hemalum and muscle fibers (M). (**F**) Basolateral region; closely packed lateral (Pl) and ubiquitary (Pu1 and Pu2) glands. Epithelium (e) and muscle fibers (M). Hemalum-eosin staining.

The foot sole is characterized by the presence of a series of ventral infoldings (Figure 4A), lined by a cylindrical epithelium. Several unicellular ventral glands (Pv) form a band that runs parallel to the epithelium. Pv cells are round, about 20 µm in diameter, with a central nucleus and a marked acidic cytoplasm, intensely stained by hemalum (Figure 4A,B). 

In a sub-epithelial position, in the sole and the basolateral region of the foot, among connectives, blood lacunae, and muscle fibers, two types of large club-shaped glands can be recognized based on their different affinity for eosin (Figure 4C). These ubiquitary glands (Pu1 and Pu2) are about 150 µm long and have a long and thin neck, crossing the epithelial cells. Glands contain a homogeneous and finely granular secretion that in Pu1 glands is intensely stained by eosin. 

In the basolateral area, large (about 250 µm) ventricose lateral glands (Pl) can be recognized (Figure 4C). Their body is localized in the sub-epithelial position, close to Pu1 and Pu2 glands, and is rich in unstained spherical vesicles of variable size (average about 5 µm) intermingled with a pale, moderately acidophilic cytoplasm. Pu1, Pu2, and Pl glands are unicellular; the crescentic nuclei are situated near the base of the glands, at the opposite side of the neck; they are surrounded by a network of muscle fibers crossing the foot in all directions and contacting the epidermis.

After exposure to DLZ, the anatomy of the foot does not change. The sole (Figure 4D) is well organized, and the infolds maintain a size and shape comparable to that of the controls. The epithelium is cylindrical and well-structured, and the gland’s shape, size, and content are unaltered (Figure 4E,F). 

**Figure 5:**
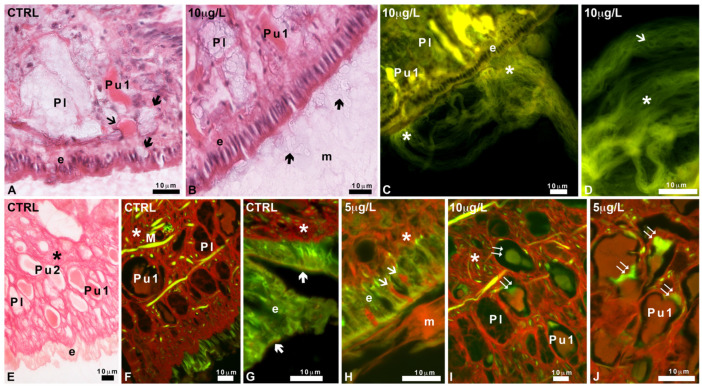
Effects of delorazepam on Planorbarius corneus pedal mucus release. (**A**) Pu1 and Pl gland necks filled with secretion (arrow and thick arrow, respectively). Epithelium (e). (**B**) Secretory vesicles (thick arrows) in mucus (m). Lateral Pl gland containing vesicles. Ubiquitary Pu1 gland with eosinophilic content. Epithelium (e). (**C**) Fibrous material (*) released from the epithelium (e). Pu1 gland necks point to the secretion site. Unstained Pl gland. (**D**) Detail of fibers released (arrow) and fibrous material (*). (**E**,**F**) Stained connectives (*) and Pu1 gland secretions. Poorly stained Pu2 and Pl glands and epithelium (e). Muscle fiber (M). (**G**) Detail of the epithelium (e) showing a moderately stained glycocalyx (thick arrows). Intensely stained connectives (*). (**H**) Intensely stained Pu1 gland necks (small arrows) and mucus (m). (**I**,**J**) Stained Pu1 gland secretions and connectives (*). Notice the presence of immature collagen, recognizable for the intense green fluorescence (double arrows). Pl glands are unstained. Hemalum-eosin staining observed under bright (**A**,**B**) or UV (**C**,**D**) light. Picrosirius red observed under bright (**E**) or UV (**F**–**J**) light.

In controls and animals exposed to DLZ 1 µg/L or 5 µg/L, secretions from Pl and Pu1 (Figure 5A) glands are visible at the level of the epithelium but not in the mucus that appears as a homogenous network (Figure 3H,I). In animals exposed to DLZ 10 µg/L, several vesicles with transparent content can be observed in the mucus, close to the epithelium (Figure 5B). For position, size, and content, these can be identified as Pl gland secretory vesicles. In the same animals, Pu1 glands release bunches of thick and intensely eosinophilic fibers (Figure 5C,D).

Picrosirius red, specific for collagen, in the controls, stains the connectives and the Pu1 gland secretions, but not the pedal epithelium (Figure 5E,F). At higher magnification, under UV light, a moderate diffused stain is observed at the apical portion of the epithelial cells, in correspondence with the glycocalyx (Figure 5G). After exposure to DLZ 1 µg/L, no significant differences are observed. 

In contrast, after exposure to DLZ 5 or 10 µg/L, the epithelium shows an increased presence of Pu1 necks filled with stained secretions; the mucus is more abundant and intensely stained (Figure 5H). While Pu1 secretions in the control group exhibit red staining (Figure 5E,F), in 5 or 10 µg/L DLZ-treated, a conspicuous green component is often present inside the gland (Figure 5I,J), indicating the presence of immature collagen.

### 2.5. Characterization of Mucus Glycoconjugates 

#### 2.5.1. Pas and Alcian Staining

**Figure 6:**
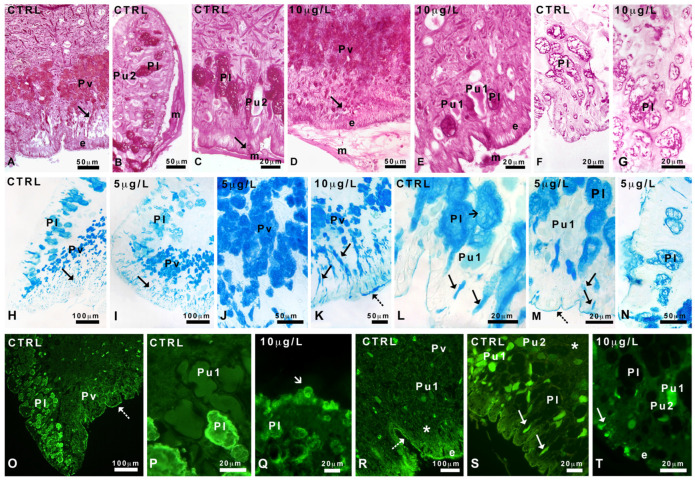
Effects of delorazepam on carbohydrate content in Planorbarius corneus pedal glands. (**A**–**C**) Stained ventral Pv gland cytoplasm and necks (arrow). Stained lateral Pl and mucus (m); poorly stained Pu2 glands. (**D**) Stained ventral Pv glands cytoplasm, necks (arrow), and mucus (m). (**E**) Stained lateral Pl gland cytoplasm and mucus (m); intensely stained ubiquitary Pu1 gland. (**F**,**G**) Detail of staining on Pl gland cytoplasm obtained after reduced exposure to the stain. (**H**,**I**) Stained ventral Pv gland cytoplasm and necks (arrow) and lateral Pl glands. (**J**) Stained ventral Pv glands. (**K**) Sole with ventral Pv gland necks filled with stained secretions (arrows). Stained glycocalyx (dot arrow). (**L**,**M**) Lateral Pl glands containing large unstained cytoplasmic vesicles (small arrow). Stained secretions are present in glands necks (arrows) and the glycocalyx (dot arrow). Unstained ubiquitary Pu1 glands. (**N**) Detail of staining on Pl gland cytoplasm but not secretory granules, visible after reduced exposure to the stain. (**O**,**P**) Stained marginal cytoplasm of lateral Pl glands and glycocalyx (dot arrow). Unstained ventral Pv and ubiquitary Pu1 glands. (**Q**) Presence of Pl secretory vesicles (small arrow) in the gland neck. (**R**) Unstained ventral Pv and ubiquitary Pu1 glands. Moderately stained glycocalyx (dot arrow). Unstained connective (*). (**S**,**T**) Intensely stained secretions in ubiquitary Pu1 glands. Moderately stained Pu2 glands and unstained lateral Pl glands. Notice the presence of labeled secretions in the gland necks (arrows). Unstained connective (*). Epithelium (e), WGA stains N-acetyl-glucosamine (glcNAc); UEA stains α-fucose. PAS staining (**A**–**G**), Alcian blue staining (**H**–**N**), WGA stains N-acetyl-glucosamine (**O**–**Q**), UEA stains α-fucose (**R**–**T**).

In control and DLZ-treated animals, no matter the concentration, PAS intensely stains the cytoplasm of ventral glands and their necks and mucus (Figure 6A–E). PAS staining is also present on the cytoplasm of Pl glands but not in the secretory granules (Figure 6F,G) visible after reduced exposure to the stain. In controls and animals exposed to DLZ 1 or 5 µg/L, Pu glands are only moderately stained (Figure 6B,C), while, after 10 µg/L treatment, the cytoplasm of ubiquitary Pu1 glands is more intensely stained (Figure 6E).

In all controls and DLZ-treated samples, Alcian blue stains the cytoplasm of the ventral glands, their neck, and the epithelial glycocalyx (Figure 6H–K). No matter the treatment, the lateral glands (Figure 6L,M) show stained cytoplasm and necks but unstained secretory vesicles (Figure 6N) after reduced exposure to the stain. The glycocalyx is also stained while the mucus is unstained (Figure 6M,N). Ubiquitary glands are unstained (Figure 6L,M).

#### 2.5.2. Staining with FITC-Lectins

In controls, the WGA stain indicates that glcNAc is present in the epithelial glycocalyx and the marginal cytoplasm of lateral Pl glands (Figure 6O,P) but not in ventral (Figure 6O) or ubiquitary (Figure 6P) glands, which are completely unstained, like the muscles and the epithelial cells. In animals exposed to DLZ, at all doses tested, glcNAc presence and localization do not change significantly. However, in 10 µg/L-treated animals, the epithelium becomes intensely fluorescent due to the presence of many surface-labeled vesicles, some of which are caught in the process of being secreted (Figure 6Q). 

In control soles, the UEA stain indicates that fucose is present in the glycocalyx (Figure 6R) but completely absent in ventral and ubiquitary glands and connectives containing the muscle fibers. In the lateral areas (Figure 6S), fucose is abundant in the Pu1 and scarce in the Pu2 glands, while lateral Pl glands and muscle fibers are entirely unstained. In feet exposed to DLZ, no significant changes in labeling distribution are observed (Figure 6T). 

Con A stain indicates that mannose is not present in control feet nor after treatment with DLZ.

### 2.6. Effects of DLZ on Foot Protein Pattern

**Figure 7:**
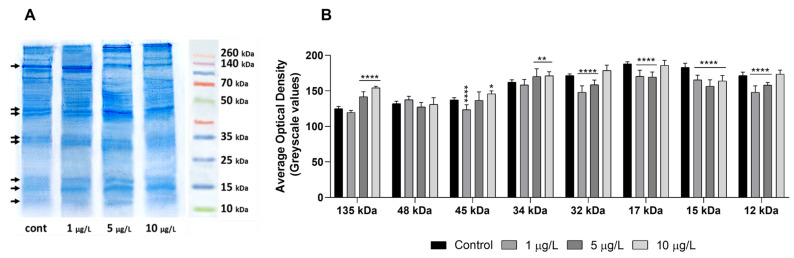
Effects of delorazepam on foot protein pattern in Planorbarius corneus. (**A**) Coomassie staining of the gel. Significant differences are present at all molecular weights (arrows). (**B**) Optical density (gray values) of the height lanes indicated in subfigure (**A**) (arrows). Grayscale values: 0 = black; 256 = white. Significant differences with respect to controls are indicated; *, *p* < 0.05; **, *p* < 0.01; ****, *p* < 0.0001.

Coomassie staining of SDS-PAGE gels (Figure 7A) demonstrates that the foot contains proteins with estimated molecular weights ranging from 15 to over 260 kDa. In the controls, the most prominent bands are at ~135 kDa, 48, and 46 kDa; two more couples of bands are observed at ~34–32 and 17–15 kDa. Several minor bands are present at middle to high molecular weights, and a smeared band is observed at about 12 kDa. 

After exposure to DLZ 1 µg/L, the bands at 135 kDa and ~17–15 kDa increase in thickness. In 5 and 10 µg/L samples, the ~135 kDa band significantly reduces, while the band at about ~48 kDa markedly increases in intensity. At 5 µg/L, in addition, the two bands at ~34–32 and ~17–15 kDa also increase in intensity, while the band at ~12 kDa becomes visible.

Optical density evaluation (Figure 7B), carried out on the above-listed eight bands, confirms the existence of significant differences among controls and treated samples.

## 3. Discussion

Locomotion in snails is greatly affected by contaminants, so much so that it is commonly used as an endpoint in toxicity testing [47,48,49,50,51]. In the case of exposure to the benzodiazepine delorazepam, we expected a depression of the motor activity [52] since GABAA-like receptors are present in mollusks [53,54], including freshwater snails (*Lymnaea stagnalis*, [55]). Results demonstrate that an opposite effect occurs in *Planorbarius*, with a dose-dependent increase of mean and mean instantaneous velocity. The reduction in V_ratio_ indicates a reduction in the exploratory activity and suggests that the treatment with DLZ caused behavioral disinhibition. The animals follow a more linear path while moving faster, reducing periodic lateral deflections from the main path [56,57]. As a consequence, the area explored reduces, decreasing the probability of intercepting traces of predators or any other possible danger. This behavior ultimately affects the timely activation of defensive mechanisms [56]. Behavioral disinhibition has also been reported in tropical snails [43] and in humans [58,59,60]. 

A decrease in feeding motivation also accompanies reduced vigilance. The lateral movements of the head are reduced, thus reducing the chance of finding food, and a high number of individuals completely stop the activity of the buccal apparatus. Further investigations are required to fully understand this response, which, in the wild, will severely impair snail survival. In particular, it will be interesting to explain why in this species, as in *Radix balthica* [18], the benzodiazepine reduces feeding, while in *Lymnaea stagnalis* [61,62], the effect is exactly the opposite. The neurotransmitter GABA is certainly implicated [63,64], but the way of action remains unclear. 

The increase in speed can be explained by the interference of DLZ in muscle contraction, a polyneuronal and polyfunctional mechanism involving different neurotransmitters, including GABA [65]. Alone or in combination [66], they are released by different nerves and may have functionally different effects [65]. Muscle anatomy and function in gastropods are poorly understood. Pedal muscles consist of smooth fibers (*Nassarius*, [67]; *Helix pomatia*, [68]), but there are also reports indicating the existence of two types of fibers (*Batillus cornutus*, [69]). In *P. corneus*, we have evidence of the presence of smooth fibers (transmission electron microscopy, that disperse, forming a tridimensional net, associated with a collagen fiber net, as indicated by Picrosirius red staining. This disposition would allow the observed rapid changes in foot length and, consequently, in instant crawling speed [70]. 

The increased speed can also be attributed to changes in mucus. Gliding in gastropods depends on the viscoelastic properties of mucus [42,43], and these allow both a glue effect with the substrate and support for the ciliary movement. In terrestrial species, mucus changes according to the role played in order to optimize muscle fibers/cilia efforts. Not much is known about the control of this dual locomotion mechanism in gastropods. In *Planorbarius,* Deliagina and Orlovsky [56] demonstrated a finely tuned control of ciliary beating, while the results obtained in this study suggest that changes in mucus composition do not accompany speed changes. Composition is reported to vary according to the species and the required degree of adhesion but also based on the presence of stressors [71]. In controls and DLZ-treated *Planorbarius corneus*, mucus stains with PAS but remains surprisingly unstained after conventional staining procedures for mucus such as Alcian blue or lectins [72,73]. Positive secretions are present in ventral, ubiquitary, and lateral glands, in their necks, and secretions are released; however, positivity is observed almost exclusively in correspondence to the glycocalyx. 

In contrast, this study indicates speed control via changes in mucus texture. In DLZ-treated animals, trails become denser and richer in vesicles and filaments, and, in histological sections, the mucus layer becomes more compact. Interestingly, this thick mucus recalls another type of mucus identified as cryptosin, produced under biological stress [74]. According to Zhong et al. [75], a rapid change in mucus characteristics is due to the release of calcium carbonate, which favors the self-assembly of glycoproteins into larger elements. In *Planorbarius*, Alcian blue staining indicates that mucins are scarce and concentrated mainly at the level of the glycocalyx. In contrast, the mucus is rich in filamentous secretions, released from Pu1 glands in great quantity after exposure to DLZ 10 µg/L, the same that induces mucus compaction. 

Both glands and secretions are poorly stained by PAS and Alcian blue but intensely stained by eosin and, more interestingly, by Picrosirius red. This suggests that filaments are proteinaceous and made of collagen [74]. Confirmation comes from the observation that in the case of extensive release, gland content, usually red, stains green with Picrosirius red, indicating the presence of newly formed, immature collagen [76]. The existence of a collagen gland in a mollusk is not a novelty, being present in mussels’ feet and involved in byssus formation [77].

Collagen is released in the form of procollagen that self-assembles in collagen fibrils, a process dependent on calcium. Based on what was reported by Zhong et al. [75], we should expect that DLZ induces a massive release of vesicles. Indeed, after treatment, this is exactly what is observed: mucus enriches in transparent vesicles that for size and content resemble those present in Pl glands. We don’t know, at the moment, the composition of these secretions, but they remained unstained with all the staining procedures tested, in accord with what is expected if they contain only water and calcium. Thanks to this coordinated release from Pu1 and Pl glands, the presence of thick filaments would increase mucus density and trailing capacity. 

The presence of collagen needs to be verified. Immunocytochemistry is in progress as well as western blotting characterization of bands observed in SDS-PAGE gels. The large band, at about 135 kDa, and the 35 kDa bands may represent unsecreted procollagen propeptides, and the observed dose-dependent variations in their intensity may be explained by the kinetics of synthesis and release. It should be pointed out that the protein pattern refers to the foot sole alone after the removal of mucus. 

Lectin staining apparently does not confirm the hypothesis: according to Soederstroem [78], collagen strands should stain with WGA and Con A and not with UEA, being rich in glucose and galactose residues and not fucose. On the other hand, it has been demonstrated that some collagen subtypes contain specific carbohydrate side chains that are still unknown, especially in invertebrates [79]. This point, therefore, will require further attention. 

## 4. Materials and Methods

### 4.1. Animal Maintenance and Treatment

Adult specimens of *Planorbarius corneus* (mean weight, 1.00 ± 0.15 g; max shell diameter, 1.46 ± 0.11 cm; N = 156) were obtained from a local pet supplier and maintained in 5 L tanks at a temperature of 20 ± 2 °C under a natural photoperiod. After two weeks of acclimation in microfiltered mineral water, pH 7.3/7.4, the animals were randomly divided into 4 groups and transferred into 0.75 L glass containers filled with 500 mL of microfiltered mineral water, pH 7.3/7.4, at a ratio of 100 mL/animal. The first group was left in the test medium (control); the other three received the test medium containing delorazepam, now one of the most (ab)used benzodiazepines by the Italian population [80], at a final concentration of 1, 5, or 10 μg/L. For the treatment, a largely consumed commercial formulation was used [20,23,81]. In the form of oral drops, it contains delorazepam at 1 mg/mL and excipients in unspecified trace quantities (96% ethanol, glycerol, glycasol N, sodium saccharin, propylene glycol). The solution was prepared by dissolving the drug directly in the water in which the animals were then placed. The exposure lasted four days and was semi-static: water was changed on the second day to remove dejections and restore benzodiazepine concentration.

### 4.2. Thigmotactic Response (TR)

**Figure 8:**
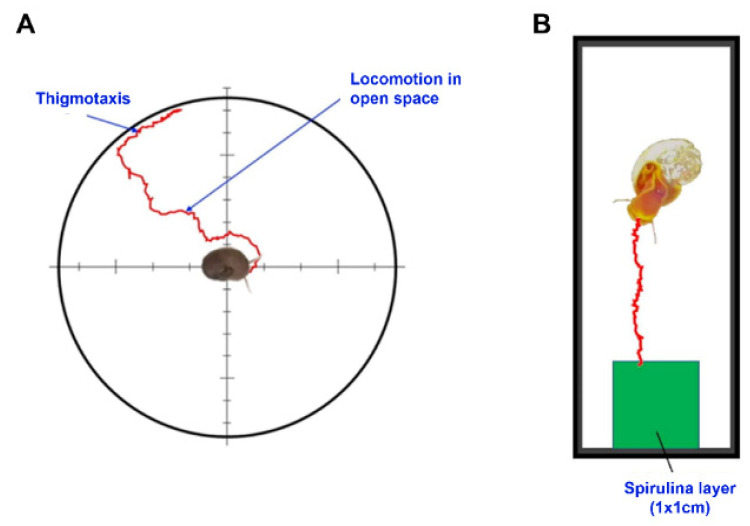
Schematic representation of the path followed by an individual. (**A**) Arena used for the thigmotactic response; two phases are distinguishable: locomotion in open space and locomotion along the wall (thigmotaxis); (**B**) the arena used a food search test.

Thigmotaxis (wall-hugging, [33]) is a conserved behavior characterized by the tendency of animals to stay close to walls or corners when exposed to novel environments, avoiding central zones (centrophobism, [34]). The test was conducted in a circular arena (Figure 8A), 15 cm in diameter, with a white background and 3 cm high black walls. The animals (n = 10 per treatment) were placed singularly in the center with a random orientation. During the manipulation, the animal retracted inside its shell, but once placed in the arena, it came out of its shell within an average of 30 s and started exploring the chamber (advancement with alternating lateral movements of the head). During manipulation, the animal retracted inside its shell, but once placed in the arena, it came out of its shell within an average of 30 s and started exploring the chamber. Only animals that were in an active state were used for the test [56]. In these conditions, the animal moved towards the wall at a relatively sustained speed, following an irregular path (Figure 8A). Upon reaching the wall, the animal maintained contact (thigmotaxis). Behavior in the arena was video recorded for at least 5 min or up to when the animal reached the wall (1080, 30 fps).

### 4.3. Food Search Test

A food search response is driven by chemosensory organs, located on the cephalic tentacles, the lips, and the buccal cavity [82]. The animal response was tested in a rectangular arena (8 × 3 × 3 cm; Figure 8B), with a transparent glass base and 3 cm high black walls. The food source consisted of a layer of dried spirulina extract, smeared onto a coverslip and placed at one extremity of the arena. Animals (n = 10 per treatment) were individually placed in the center of the arena, oriented toward the food. During manipulation, the animal retracted inside its shell, but once placed in the arena, it came out of its shell within an average of 30 s and started exploring the chamber, displaying feeding behavior (rhythmic movements of the buccal apparatus, [57]). Only animals that were in an active state were used for the test [56]. Video recording was performed until the animal reached the food or for a maximum of 5 min if the animal did not reach the goal.

### 4.4. Video Analysis and Speed Determination

For thigmotaxis, the camera was placed above the arena, while for the food search test, it was placed below to record mouth movements. The videos were analyzed with Tracker (Open-Source Physics, Version 6.0.6, 2021), determining at 1 s (30 frames) intervals the instantaneous speed (in cm/min), from which we determined the average instantaneous speed (V_mi_). The average speed (V_m_) was also determined. In the thigmotactic test, V_mi_ was determined only during open-space locomotion. V_m_ was instead calculated both during locomotion in open space (V_om_ = the ratio between the distance from the center of the arena and the point at which the animal first touches the wall and the time the animal took to reach the wall) and during thigmotaxis (Vtm). In the food research test, V_m_ was calculated as a ratio between the linear distance from the initial position of the animal and the spirulina layer and the total time taken to reach the food, or the maximal linear distance reached by the animal within 5 min (if it did not reach the goal). In both tests, V_ratio_ (i.e., the ratio between V_mi_ and V_m_ during the open space locomotion in the thigmotactic test and between V_mi_ and V_m_ in the food research test), which is an index of the tortuosity of the path followed by the animal to reach the goal (arena wall or spirulina layer), was calculated. Finally, the frequency of rhythmic opening and radula movement of the buccal apparatus (the number of openings per min, Fb) was determined in the food search test and used as an index of animals’ feeding activity.

### 4.5. Mucus Collection

On the fourth day of treatment, ten controls and ten animals treated with 10 µg/L DLZ were placed in the 3 × 3 × 8 cm chamber containing water or water and DLZ. Object slides, previously washed in ethanol and distilled water, were placed on the floor of the chamber. The animals were allowed to glide on the slides for 20 min. The slides with the mucus trails were then collected, air-dried to allow the mucus to adhere to the glass, and fixed in 95% ethanol for 3 min. After air drying, the slides were examined under a phase contrast microscope or stained with eosin (0.25% for a few seconds).

### 4.6. Histological Analysis

Feet were dissected from twenty animals (n = 5 per treatment) previously cooled to 4 °C for 10 min to minimize neural activity [83]. Tissue samples were fixed in 5 mL Bouin’s solution (picric acid, formaldehyde, acetic acid; 15:5:1 *v*/*v*/*v*), dehydrated in graded ethanol, and embedded in paraffin according to routine protocols [84]. Sections (6 μm) were stained with hemalum-eosin to show general morphology or with periodic acid-Schiff (PAS) or Alcian blue to highlight mucin, glycogen, and glycoproteins [23]. Carbohydrate residues were characterized by staining with a panel of lectins: WGA specific for N-acetyl-glucosamine, UEA-1 specific for α-fucose, and Con A specific for mannose (Vector Laboratories Inc, Newark, NY, USA; 2 mg/mL). Briefly, 1 μL of lectin was diluted in 30 μL of PBS, and the solution was placed on the tissue slice, in the dark, in a humid chamber. After 15 min, the section was rinsed in PBS, mounted with a drop of buffer, and observed under UV (maximum excitation at 495 nm and maximum emission at 515 nm). Negative controls were prepared by incubating slides in the presence of the specific competing sugar or by omitting the lectin [72]. To highlight collagen, sections were stained with Picrosirius red (0.1 g Direct Red 80 from Merck was dissolved in saturated picric acid) and observed under fluorescent light (emission at 561 nm, detection with a 635 to 685 nm bandpass filter; [85]). Under these conditions, mature collagen emits in red, while immature collagen emits in green. All the images were acquired with a Zeiss Axiocam camera applied to a Zeiss Axioskop microscope (Zeiss, Jena, Germany).

### 4.7. Protein Analysis by SDS-PAGE

Foot samples from three different animals per treatment were collected, rapidly dried by repeated contact with a clean towel to reduce pedal mucus, frozen, and stored at −80 °C. For protein extraction, the tissue was dissolved in 50 mM PBS, pH 7.5, containing a protease inhibitor cocktail (Sigma, St. Louis, MI, USA), sonicated for 2.5 min, and centrifuged at 18.800 g for 20 min [86]. The supernatant was collected, and the extracted proteins (30 μg) were run on a 12.5% SDS/polyacrylamide gel, in tris-glycine buffer, at 60 mA and stained with Coomassie Blue R-250 [84]. Analyses were carried out in triplicate. 

The protein pattern was examined by adapting the procedure described by Vincent et al. [87]. Briefly, after staining, Coomassie gels were scanned at high resolution (400 dpi) to produce 8-bit, 256 grayscale images that were saved in TIFF format. Images were analyzed using the Image J program (free version 1.8.0; last update 22 May 2023). Band density was determined and converted into optical density (grayscale) using the Plot Profile option.

### 4.8. Statistical Analysis

Data were processed with GraphPad-Prism 8 software (GraphPad Software, Inc., San Diego, CA, USA). Results obtained from behavioral tests were reported as means ± SE, and differences were analyzed for significance by one-way or two-way ANOVA, followed by the post hoc Sidak test (*p* < 0.05). For the protein pattern, the numerical data obtained were saved and used to calculate means ± SD and to draw the graph. The significance of differences was tested by ANOVA followed by Bonferroni’s test. 

## 5. Conclusions

In conclusion, in *Planorbarius corneus*, DLZ induces hyperactivity and behavioral disinhibition and impairs feeding, effects that severely impact animal fitness and, eventually, survival. Gastropods are important organisms in the balance of aquatic ecosystems, thanks to their over 4000 species. Most of these live in shallow fresh or coastal waters, the most endangered by anthropic contamination, and, therefore, better knowledge of responses will help in managing long-term environmental impacts. The increase in locomotory speed is accompanied by a significant compaction of the mucus, due to a massive release from the ubiquitary Pu1 glands of a proteinaceous fibrillar material. At the moment, the evidence indicates that this is composed of collagen and that its assembly is directed by secretions released by a lateral Pl gland. Snail mucus has found application in cosmetics and medicine, as a wound healer, thanks to the presence of collagen. The evidence collected in *Planorbarius*, a small model snail, could prompt the search for other, commercially more convenient species to facilitate large-scale exploitation of this natural resource.

## Data Availability

Data is contained within the article
